# Cognitive frailty in relation to vitamin B12 and 25-hydroxyvitamin D in an elderly population: a cross-sectional study from NHANES

**DOI:** 10.3389/fnut.2024.1430722

**Published:** 2024-08-27

**Authors:** Yu Pan, Xue Yin Tang, Juan Yang, Zhu Qing Feng, Yan Yuan, Yi Jiang, Gui Ming Hu, Jiang Chuan Dong

**Affiliations:** ^1^Department of Geriatrics, The Second Affiliated Hospital of Chongqing Medical University, Chongqing, China; ^2^Department of Integrated of Chinese and Western Medicine, The First Affiliated Hospital of Chongqing Medical University, Chongqing, China; ^3^Department of Integrated of Chinese and Western Medicine, The Second Affiliated Hospital of Chongqing Medical University, Chongqing, China

**Keywords:** cognitive frailty, 25-hydroxyvitamin D, vitamin B12, cross-sectional study, NHANES

## Abstract

**Background:**

Nutritional support has been identified as a potential intervention for cognitive frailty; however, the association between 25-hydroxyvitamin D [25-(OH)D], vitamin B12, and cognitive frailty remains ambiguous.

**Methods:**

This study utilized data from two cycles (2011–2012, 2013–2014) of the National Health and Nutrition Examination Survey (NHANES) to investigate this relationship. The researchers constructed a 41-item frailty index encompassing diverse aspects of physical functioning, psychological evaluation, and medical conditions, and evaluated each participant individually. The study utilized Spearman's rank correlation coefficient and univariate ordered logistic regression to assess the relationships between variables and cognitive frailty. Recursive feature elimination and cross-validation methods were employed to identify the most influential variables for building and optimizing multivariate ordered logistic regression models. Subgroup analyses and interaction tests were further conducted to validate the identified correlations.

**Results:**

The findings of this study confirm a negative linear correlation between 25-(OH)D levels and cognitive frailty in older adults. Specifically, a one-unit increase in 25-(OH)D levels was associated with a 12% reduction in the risk of cognitive frailty. The result was further supported by subgroup analyses and interaction tests.

**Conclusion:**

The existence of a negatively correlated linear association between 25-(OH)D levels and cognitive frailty in older adults is plausible, but further rigorously designed longitudinal studies are necessary to validate this relationship.

## 1 Introduction

Frailty is a prevalent clinical syndrome among older adults, marked by susceptibility and diminished capacity for normal physiological functions when faced with acute stress (1). The most commonly recognized manifestation of frailty is a specific physical phenotype outlined by Fried, which encompasses the complex interplay of physiological functioning in older individuals, including unintentional weight loss, weakness, poor endurance, and energy, slowness of movement, low grip strength, reduced levels of physical activity (2). However, it is now widely acknowledged that this assessment of physical frailty may not fully capture the complexity of frailty, particularly cognitive functioning (3). Recent research has incorporated cognitive functioning into frailty assessments, leading to the emergence of cognitive frailty as a distinct clinical entity characterized by the simultaneous presence of physical frailty and cognitive impairment that has not yet progressed to dementia (4, 5). It is unequivocal that cognitive impairment is closely associated with frailty in a clinical context. Epidemiological studies have demonstrated that frailty is linked to a heightened likelihood of cognitive decline in the future, whereas cognitive impairment may elevate the risk of developing frailty (6, 7). A particular study delved into the causal connection between frailty and cognitive impairment, as well as examined the interplay between these two conditions, thereby enhancing the understanding of cognitive frailty (8). For instance, a research study examining elderly Chinese males found that individuals with cognitive impairment were at a higher risk of developing physical frailty after 4 years compared to those without cognitive impairment (9). Nevertheless, another multicenter study involving community-dwelling older adults in Chicago revealed that older adults with physical frailty were more susceptible to cognitive impairment, which subsequently led to an escalation in somatic frailty (10).

While cognitive frailty has not advanced to dementia, the transition from cognitive decline to dementia is a continuous and irreversible process for which there is currently no effective treatment. Current interventions primarily aim to mitigate the risk of cognitive decline by addressing potential risk factors (11). Although certain factors may be beyond modification, others such as excessive alcohol consumption, smoking, obesity, lack of physical activity, and dietary or nutritional deficiencies can be controlled to decrease the occurrence of cognitive deficits or impede their advancement (11, 12). Therefore, nutritional supplementation may offer a potential avenue for both preventing and treating cognitive frailty (13, 14).

25-hydroxyvitamin D [25-(OH)D] and vitamin B12 are essential organic compounds that are crucial for the proper functioning of the body's physiology and are involved in key metabolic pathways that support fundamental cellular processes (15). Nevertheless, due to their limited endogenous synthesis in the elderly population, regular dietary supplementation of vitamin B12 and 25-(OH)D is necessary (16). Regrettably, the potential impact of these vitamins on cognitive frailty remains unrecognized, as previous research has primarily examined their potential to enhance cognitive function without considering potential somatic frailty effects.

To address this research deficiency, a 41-item Frailty Index (FI) scale was developed in accordance with established standard procedures for FI construction, encompassing various dimensions of psychological and physical health (17). Subsequently, utilizing publicly available datasets, a combination of conventional statistical techniques and machine learning algorithms was employed to explore the associations between cognitive frailty and levels of vitamin B12 and 25-(OH)D, with the overarching aim of offering a nutritional perspective for the management of cognitive frailty.

## 2 Methods

### 2.1 Study design

The National Health and Nutrition Examination Survey (NHANES) is a research program aimed at evaluating the health and nutritional wellbeing of individuals residing in the United States. Utilizing a blend of questionnaires and physical assessments, the program focuses on particular demographic groups or health-related concerns. The NHANES study methodology received approval from the Ethics Review Board of the National Center for Health Statistics at the U.S. Centers for Disease Control and Prevention, with all participants providing written informed consent before participation in the survey.

**Figure 1:**
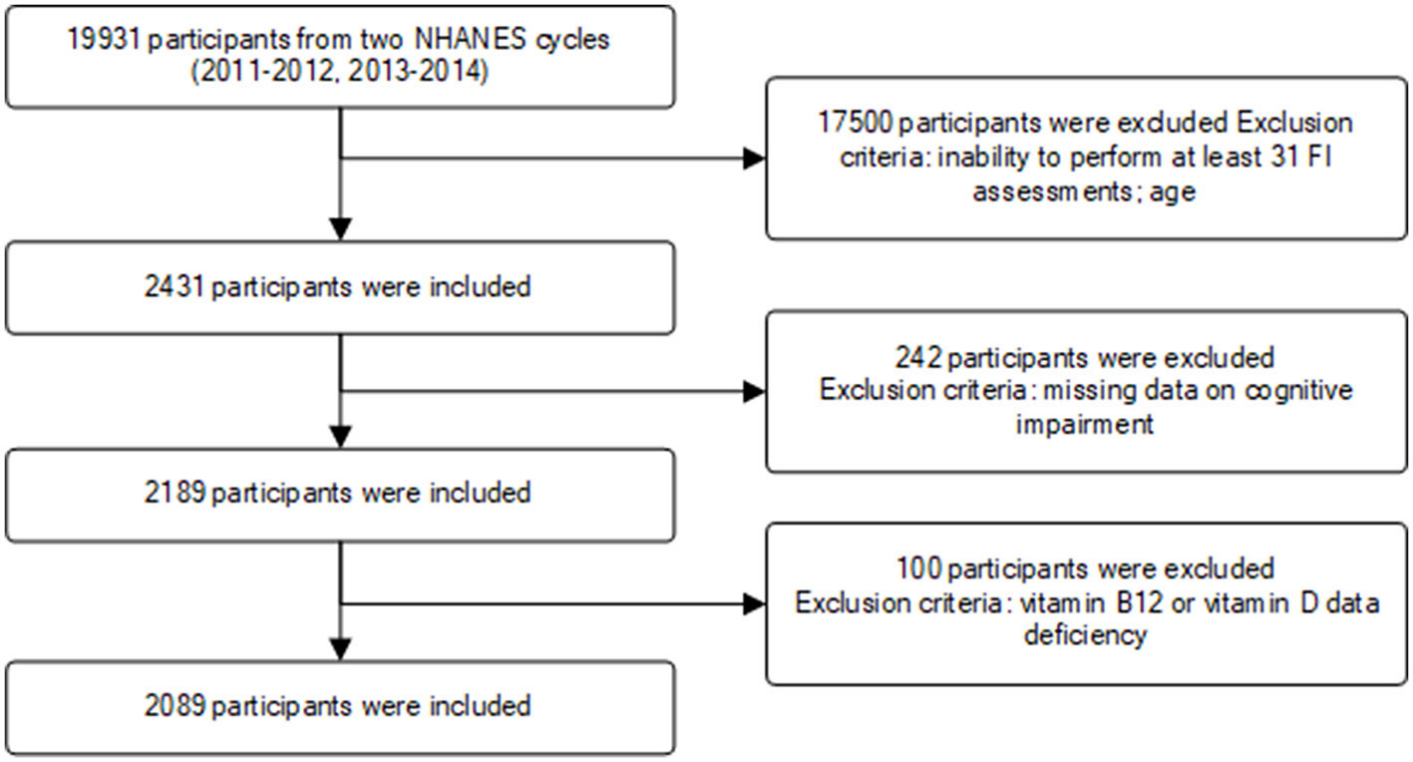
The participants' selection process.

This study incorporated data from two NHANES survey cycles (2011–2012, 2013–2014). A total of 19,931 participants were initially enrolled, with exclusions made for individuals with missing relevant data or those under the age of 65. Ultimately, 2,089 participants met the inclusion criteria for the study (Figure 1).

### 2.2 Frailty assessment

Employing established criteria and methodologies for constructing the Frailty Index (FI) and utilizing data from the NHANES (17), this study developed a 41-item assessment scale to evaluate an individual's frailty status. The scale was designed to comprehensively evaluate various aspects of health, illness, physical functioning, and mental health in order to determine the level of frailty in each participant. By applying the FI scale and referencing existing research, the study further classified frailty into three categories: absence of frailty (FI ≥ 0.12), pre-frailty (0.12 <FI <0.25), and frailty (FI ≤ 0.25) (17, 18). The item composition and scoring criteria of the FI scale are detailed in Appendix 1.

### 2.3 Cognitive frailty

Currently, there is no gold standard for identifying low cognitive performance on the Consortium to Establish a Registry for Alzheimer's Disease (CERAD), Animal Fluency Test (AFT), and the Digit Symbol Substitution Test (DSST). Consequently, we employed the 25th percentile of scores, or the lowest quartile, as the threshold, aligning with the methodological approach used in existing research (19). Furthermore, given that all study participants fell within the 65–80 age range, scores were stratified by age groups (65–72 years, 73–80 years) to address the known impact of age on cognitive functioning (20). The CERAD test established cutoff values of 22 and 18 for low cognitive performance across the two age groups, while the AFT and DSST had cutoff values of 13, 11, and 34, 28, respectively. Participants were categorized into either the low cognitive performance group, with scores falling below the specified cutoff values, or the normal cognitive performance group based on their performance in each dimension.

Furthermore, given the bidirectional negative relationship between cognitive impairment and somatic frailty, wherein cognitive impairment significantly heightens the likelihood of somatic frailty and vice versa, individuals with either somatic frailty or cognitive impairment not yet advanced to cognitive frailty were classified as pre-cognitive frail in this study. Cognitive frailty was then categorized as non-cognitive frail, pre-cognitive frail, and cognitive frail.

### 2.4 25-(OH)D and vitamin B12

According to current guidelines, total serum 25-hydroxy-25-(OH)D (25-(OH)D) is recommended as a suitable biomarker for evaluating 25-(OH)D status, enabling the identification of potential deficiencies or excessive levels of the 25-(OH)D (21). A total serum 25-(OH)D level below 20 ng/mL (<50 nmol/L) may indicate deficiency, while levels between 20–29 ng/mL (50–72.5 nmol/L) are considered insufficient in the absence of evident clinical symptoms (22, 23). Participants with concentrations >30 ng/ml (50 nmol/L) were considered as normal 25-(OH)D status.

Furthermore, the study population was stratified based on distinct serum B12 categories: (1) deficiency, characterized by serum B12 levels <140 pmol/L; (2) insufficiency, with serum B12 levels ranging from 140 to 300 pmol/L; (3) normal levels, falling within the range of 300 to 700 pmol/L; and (4) elevation, denoted by serum B12 concentrations exceeding 700 pmol/L (24).

### 2.5 Ethics statement

Studies involving human subjects were reviewed and approved by protocols used by NHANES, approved by the Research Ethics Review Board of the National Center for Health Statistics, and written informed consent was provided by all participants, with information available on the official NHANES website (https://wwwn.cdc.gov/nchs/nhanes/index.htm). Data from the NHANES survey are publicly available, and all participants provided written informed consent to participate in the NHANES study. All patient information in the database used for this study was anonymized, and all participants were aware of and consented to the data collection activities. No further ethical approval or informed consent was required for this study.

### 2.6 Statistical analysis

This study employed a hybrid approach involving statistical analysis and machine learning methodologies to thoroughly examine the association between 25-(OH)D, vitamin B12, and cognitive frailty. Initially, the study characterized the dataset through descriptive statistics and subsequently investigated the correlation between 25-(OH)D, vitamin B12, and cognitive frailty utilizing Spearman's rank correlation coefficient (25). Furthermore, chi-square tests were utilized to investigate the relationships between various categorical variables and cognitive frailty. Subsequently, the impact of relevant variables on the likelihood of cognitive frailty was thoroughly examined through univariate ordered logistic regression. To address potential issues of covariance among variables, the study employed the variable inflation factor (VIF) for identification and selected key variables using recursive feature elimination and cross-validation (RFECV) (26). In sensitivity analyses, vitamins implicated in cognitive frailty were transformed into categorical variables to further elucidate the relationship between their levels and cognitive frailty, as well as their statistical significance. Subgroup analyses were conducted to explore the interaction between these vitamins and demographic characteristics. Moreover, random forest modeling was utilized to evaluate the significance of each variable, identifying key biomarkers that significantly impacted cognitive frailty. This comprehensive analytical approach, utilizing multiple levels and techniques, enhances comprehension of the impact of vitamins on cognitive health in elderly individuals and establishes a robust scientific foundation for the formulation of prevention and intervention strategies aimed at addressing cognitive frailty.

## 3 Results

### 3.1 Participant characteristics

This study included a total of 2089 individuals, all aged between 65 and 80 years, aligning with the criteria set forth by the World Health Organization and the United Nations for defining older adults. The mean age of the sample was 73 years (SD = 5), with females comprising 51% (*n* = 1,061) of the participants. A majority of the subjects were married (56%) and the subject population was predominantly the White race (54%). Education levels varied significantly within the sample, with nearly half (48%) having received higher education (Table 1). The results of the chi-square test indicated significant correlations between ethnicity, education, and marital status with cognitive frailty, while gender was not. Analysis of blood test results revealed correlations between most variables and cognitive frailty, with the exceptions of platelet and red blood cell folate. Calculations showed that Spearman rank correlation coefficients, which range from −1 to +1, where +1 indicates a perfect positive correlation, −1 indicates a perfect negative correlation, and 0 indicates no correlation (25). Noteworthy findings include positive correlations between uric acid, glycosylated hemoglobin, blood glucose, blood creatinine, blood urea nitrogen (BUN), and leukocytes with cognitive frailty, while negative correlations were observed between total cholesterol, hemoglobin, mean corpuscular volume (MCV), and hematocrit (HCT) with cognitive frailty. Additionally, 25-(OH)D exhibited a negative association with cognitive frailty, while vitamin B12 did not show such a relationship. Consequently, subsequent analyses were focused solely on the relationship between 25-(OH)D and cognitive frailty.

**Table 1:** Variables and baseline characteristics of the participants.

**Variables**		**Total**	**Non- cognitive frail**	**Pre- cognitive frail**	**Cognitive frail**	***P*-value**	**Spearman correlation coefficient**
Age		73 ± 5	72 ± 5	73 ± 5	74 ± 5	<0.001	0.19
Total cholesterol (mg/dL)		187 ± 40	192 ± 40	188 ± 41	177 ± 39	<0.001	−0.14
WBC (10^9^/L)		7.0 ± 1.9	6.7 ± 1.9	7.0 ± 2.0	7.2 ± 1.9	<0.001	0.11
Hemoglobin (g/dL)		14 ± 1.4	14 ± 1.2	14 ± 1.4	13 ± 1.5	<0.001	−0.20
MCV (fL)		91 ± 5.4	91 ± 4.7	91 ± 5.6	90 ± 5.7	0.03	−0.05
HCT (%)		40 ± 4	41 ± 3.6	40 ± 3.9	39 ± 4.4	<0.001	−0.18
PLT (10^9^/L)		218 ± 56	219 ± 52	217 ± 56	221 ± 61	0.9	0.002
BUN (mg/dL)		17 ± 7	16 ± 5.8	17 ± 6.8	19 ± 8.3	<0.001	0.11
Glucose, serum (mg/dL)		112 ± 37	105 ± 28	113 ± 37	121 ± 46	<0.001	0.14
Creatinine, serum (mg/dL)		1 ± 0.4	1 ± 0.3	1 ± 0.4	1 ± 0.5	<0.001	0.15
Glycosylated hemoglobin (%)		6 ± 1	5.8 ± 0.7	6 ± 0.9	6 ± 1	<0.001	0.19
Uric acid (mg/dL)		5.8 ± 1.4	5.6 ± 1.3	5.7 ± 1.4	6 ± 1.5	<0.001	0.08
Vitamin B12 (pmol/L)		540 ± 675	540 ± 516	548 ± 888	528 ± 465	0.3	−0.03
25-(OH)D (nmol/L)		79 ± 32	83 ± 32	78 ± 30	75 ± 33	<0.001	−0.09
Erythrocyte folate (nmol/L)		1,427 ± 684	1,428 ± 629	1,434 ± 691	1,412 ± 745	0.07	−0.04
Gender						0.3	
	Male	1,016 (49%)	365 (50%)	405 (50%)	246 (46%)		
	Female	1,061(51%)	372 (50%)	400 (50%)	289 (54%)		
Race						<0.001	
	Asian	169 (8.1%)	72 (9.8%)	69 (8.6%)	28 (5.2%)		
	African-American	423 (20%)	112 (15%)	170 (21%)	141 (26%)		
	The White race	1,113 (54%)	471 (64%)	407 (51%)	235 (44%)		
	Latino/Hispanic	339 (16%)	72 (9.8%)	145 (18%)	122 (23%)		
	Other race	33 (1.6%)	10 (1.4%)	14 (1.7%)	9 (1.7%)		
Education						<0.001	
	Primary education and below	591 (28%)	91 (12%)	221 (28%)	79 (52%)		
	Secondary education	482 (23%)	167 (23%)	194 (24%)	121 (23%)		
	Higher education	1,002 (48%)	479 (65%)	388 (48%)	135 (25%)		
Marital status						<0.001	
	Unmarried	90 (4.3%)	28 (3.8%)	39 (4.9%)	23 (4.3%)		
	Married	1,162 (56%)	474 (64%)	445 (55%)	243 (46%)		
	Widowed	525 (25%)	130 (18%)	204 (25%)	191 (36%)		
	Divorced	299 (14%)	105 (14%)	117 (15%)	77 (14%)		

Gender, race, education, and marital status were expressed as percentages. *P* values for categorical variables were calculated using the chi-square test. *P* values for continuous variables were calculated using Spearman's rank correlation coefficient method. Mean ± standard deviation: age, total cholesterol (mg/dL), WBC (10^9^/L), hemoglobin (g/dL), mean corpuscular volume (MCV) (fL), hematocrit (HCT) (%), PLT (10^9^/L), urea nitrogen (BUN) (mg/dL), serum glucose (mg/dL), serum creatinine (mg/dL), glycosylated hemoglobin (%), uric acid (mg/dL), vitamin B12 (pmol/L), 25-(OH)D (nmol/L), erythrocyte folate (nmol/L). WBC, white blood cell count; MCV, mean corpuscular volume; HCT, hematocrit; PLT, platelet count; BUN, urea nitrogen.

### 3.2 Univariate ordered logistic regression

Univariate ordered logistic regression analyses were performed to independently evaluate potential relationships between each variable. The findings of the univariate ordered logistic regression analysis for cognitive frailty are presented in Table 2. Notably, a significant correlation was observed between cognitive frailty and 25-(OH)D levels among older individuals (*P* < 0.001), while no such correlation was found with other vitamins such as vitamin B12 and folic acid. In addition, the majority of blood test-related variables exhibited independent associations with cognitive frailty, which warrants further investigation.

**Table 2:** Univariate ordered logistic regression analysis for cognitive frailty.

**Variables**	**Coefficient**	**OR (95% CI)**	***P*-value**
Age	0.07	1.07 (1.05, 1.08)	<**0.001**
Total cholesterol	−0.006	0.994 (0.992, 0.996)	<**0.001**
**WBC**	0.09	1.09 (1.05, 1.14)	<**0.001**
**MCV**	−0.02	0.978 (0.963, 0.992)	**0.003**
PLT	<0.001	1.0 (0.999, 1.0)	**0.6**
BUN	0.04	1.0 (1.0, 1.1)	**<0.001**
Glucose, serum	0.008	1.0 (1.0, 1.0)	**<0.001**
Creatinine, serum	0.8	2.3 (1.8, 2.8)	**<0.001**
Glycosylated hemoglobin	0.4	1.5 (1.4, 1.6)	**<0.001**
Uric acid	0.1	1.1 (1.1, 1.2)	**<0.001**
HCT	−0.09	0.92 (0.90, 0.93)	**<0.001**
Hemoglobin	−0.3	0.8 (0.7, 0.8)	**<0.001**
Vitamin B12	−0.00001	1.0 (1.0, 1.0)	**0.08**
25-(OH)D	−0.006	0.994 (0.992, 0.997)	**<0.001**
Erythrocyte folate	−0.00002	1.0 (1.0, 1.0)	**0.7**
**Gender**			
Male		***Reference***	
Female	0.09	1.1 (0.9, 1.3)	**0.3**
**Education**			
Primary education and below		***Reference***	
Secondary education	−1.0	0.4 (0.3, 0.4)	<**0.001**
Higher education	−1.7	0.2 (0.2, 0.2)	<**0.001**
**Marital status**			
Married		***Reference***	
Unmarried	0.4	1.4 (1.0, 2.1)	**0.08**
Divorced	0.3	1.3 (1.0, 1.6)	**0.04**
Widowed	0.8	2.1 (1.8, 2.6)	<**0.001**
**Race**			
Asian		***Reference***	
African-American	0.8	2.2 (1.6, 3.0)	**<0.001**
The White race	0.1	1.1 (0.8, 1.5)	**0.5**
Latino/Hispanic	1.0	2.6 (1.9, 3.7)	**<0.001**
Other race	0.6	1.7 (0.9, 3.4)	**0.1**

Results of univariate ordered logistic regression analysis.

Among the categorical variables, one group was designated as the reference. The findings indicated that gender, marital status, White race, and unrepresented races did not demonstrate a statistically significant correlation with cognitive frailty. Conversely, individuals who were widowed, African-American, and Latino/Hispanic were potentially more susceptible to cognitive frailty progression.

### 3.3 Linear relationship

**Figure 2:**
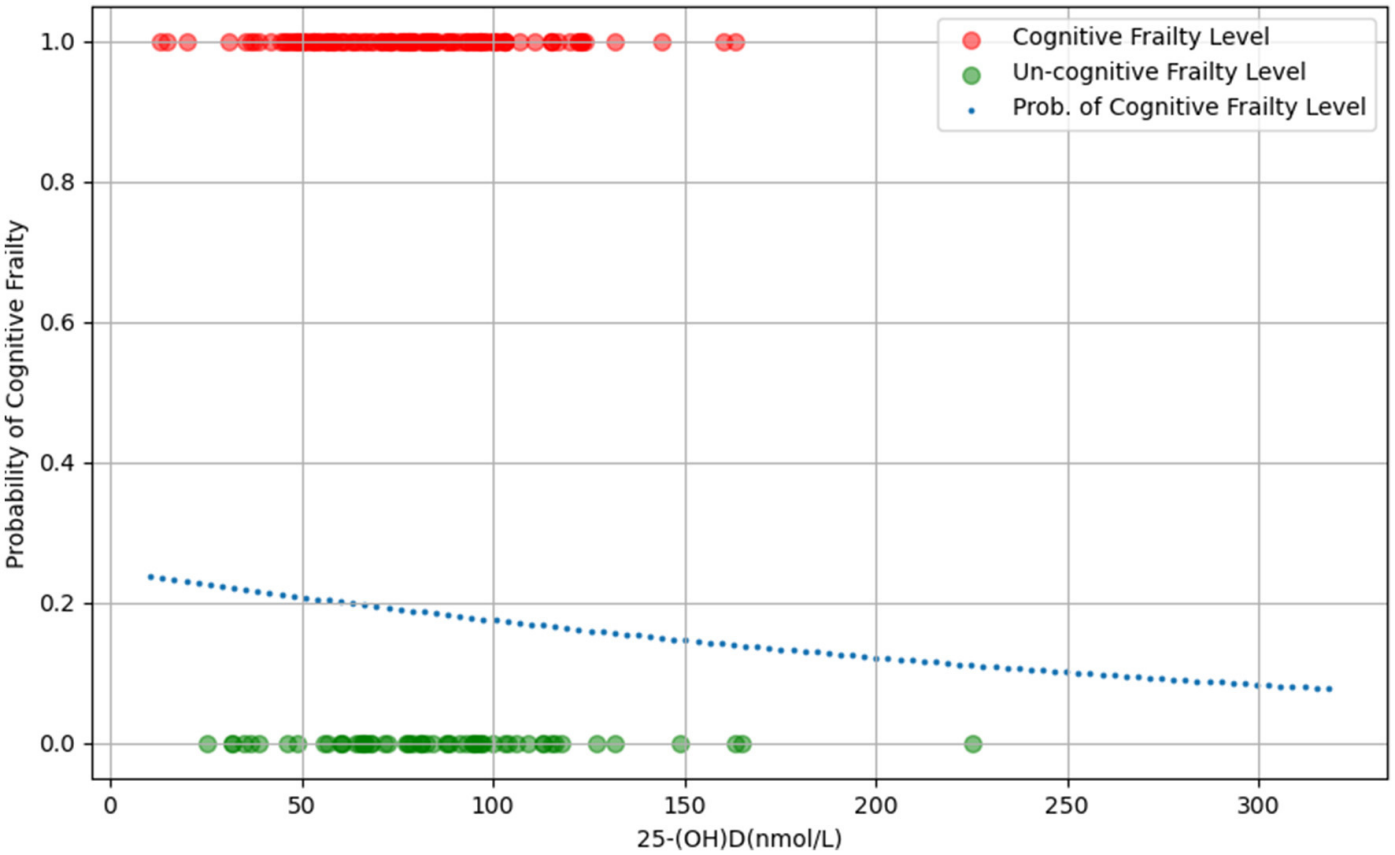
The scatter plot of the relationship between 25-(OH)D levels and probability of cognitive frailty.

The findings from the univariate ordered logistic regression analysis and Spearman's rank correlation coefficients indicate a positive linear association between 25-(OH)D levels and cognitive frailty in the elderly, suggesting that higher levels of 25-(OH)D are associated with a reduced likelihood of cognitive frailty (Figure 2).

### 3.4 Multivariable adjusted regression models

Before conducting multivariate ordered logistic regression analyses, VIF was utilized to identify and address potential covariance issues among the variables. HCT (VIF>10) was excluded to prevent any potential interference as a covariate (Supplementary Table 1). Following this, the most impactful feature variables were chosen using the RFECV technique to enhance the model's performance. This process resulted in the exclusion of two variables, uric acid and BUN. Table 3 presents the findings of the multivariate logistic regression analysis examining the association between 25-(OH)D levels, other pertinent variables, and cognitive frailty. Following comprehensive adjustment for uric acid, BUN, and HCT variables, a statistically significant inverse relationship between 25-(OH)D levels and cognitive frailty was observed, aligning with the outcomes of univariate ordered logistic regression. Subsequent calculations indicated that a one-unit increase in 25-(OH)D levels corresponded to a 12% decrease in the likelihood of experiencing cognitive frailty.

**Table 3:** Multivariable regression models.

**Variables**	**Model I**			**Model II**		
	**Coefficient**	**OR**	***P*-value**	**Coefficient**	**OR**	***P*-value**
25-(OH)D	−0.1	0.9 (0.8, 1.0)	0.05	−0.1	0.9 (0.8, 1.0)	0.03
Erythrocyte folate	−0.1	1.0 (0.8, 1.0)	0.4	−0.05	1.0 (0.9, 1.1)	0.4
Age	0.4	1.5 (1.4, 1.7)	<0.001	0.4	1.5 (1.3, 1.7)	0.00
Total cholesterol	−0.2	0.8 (0.7, 0.9)	0.001	−0.2	0.8 (0.7, 0.9)	0.001
WBC	0.1	1.1 (1.0, 1.3)	0.04	0.1	1.1 (1.0, 1.3)	0.04
MCV	0.007	1.0 (0.9, 1.1)	0.9	−0.002	1.0 (0.9, 1.1)	0.99
PLT	0.009	1.0 (0.9, 1.1)	0.9	0.01	1.0 (0.9, 1.1)	0.9
BUN	−0.04	1.0 (0.8, 1.1)	0.6			
Glucose, serum	0.06	1.1 (0.9, 1.2)	0.4	0.06	1.1 (0.9, 1.2)	0.4
Creatinine, serum	0.1	1.1 (1.0, 1.3)	0.2	0.1	1.1 (1.0, 1.2)	0.1
Glycosylated hemoglobin	0.2	1.3 (1.1, 1.5)	0.003	0.2	1.3 (1.1, 1.5)	0.003
Uric acid	0.1	1.1 (0.9, 1.2)	0.5			
Hemoglobin	−0.1	0.9 (0.8, 1.0)	0.08	−0.1	0.9 (0.8, 1.0)	0.1
Female	0.1	1.2 (0.9, 1.5)	0.3	0.1	1.1 (0.9, 1.5)	0.4
Secondary education	−0.9	0.4 (0.3, 0.5)	<0.001	−0.9	0.4 (0.3, 0.5)	<0.001
Higher education	−1.4	0.2 (0.2, 0.3)	<0.001	−1.5	0.2 (0.2, 0.3)	<0.001
Unmarried	0.2	1.2 (0.7, 2.0)	0.6	0.2	1.2 (0.7, 1.9)	0.6
Divorced	0.1	1.2 (0.8, 1.6)	0.4	0.2	1.2 (0.8, 1.6)	0.4
Widowed	0.2	1.2 (0.9, 1.6)	0.1	0.2	1.2 (1.0, 1.6)	0.1
African-American	0.4	1.5 (0.9, 2.4)	0.09	0.4	1.5 (1.0, 2.4)	0.07
The White race	−0.2	0.9 (0.6, 1.3)	0.5	−0.2	0.9 (0.6, 1.3)	0.5
Latino/Hispanic	0.5	1.7 (1.1, 2.7)	0.02	0.5	1.7 (1.1, 2.6)	0.03
Other race	−0.2	0.8 (0.3, 2.2)	0.7	−0.2	0.8 (0.3, 2.3)	0.7

Results: OR (95%CI) *P*-value/Outcome: Cognitive frailty; Model I adjusted for: Variables except HCT. Model II adjusted for: Variables except HCT, uric acid, and BUN.

**Figure 3:**
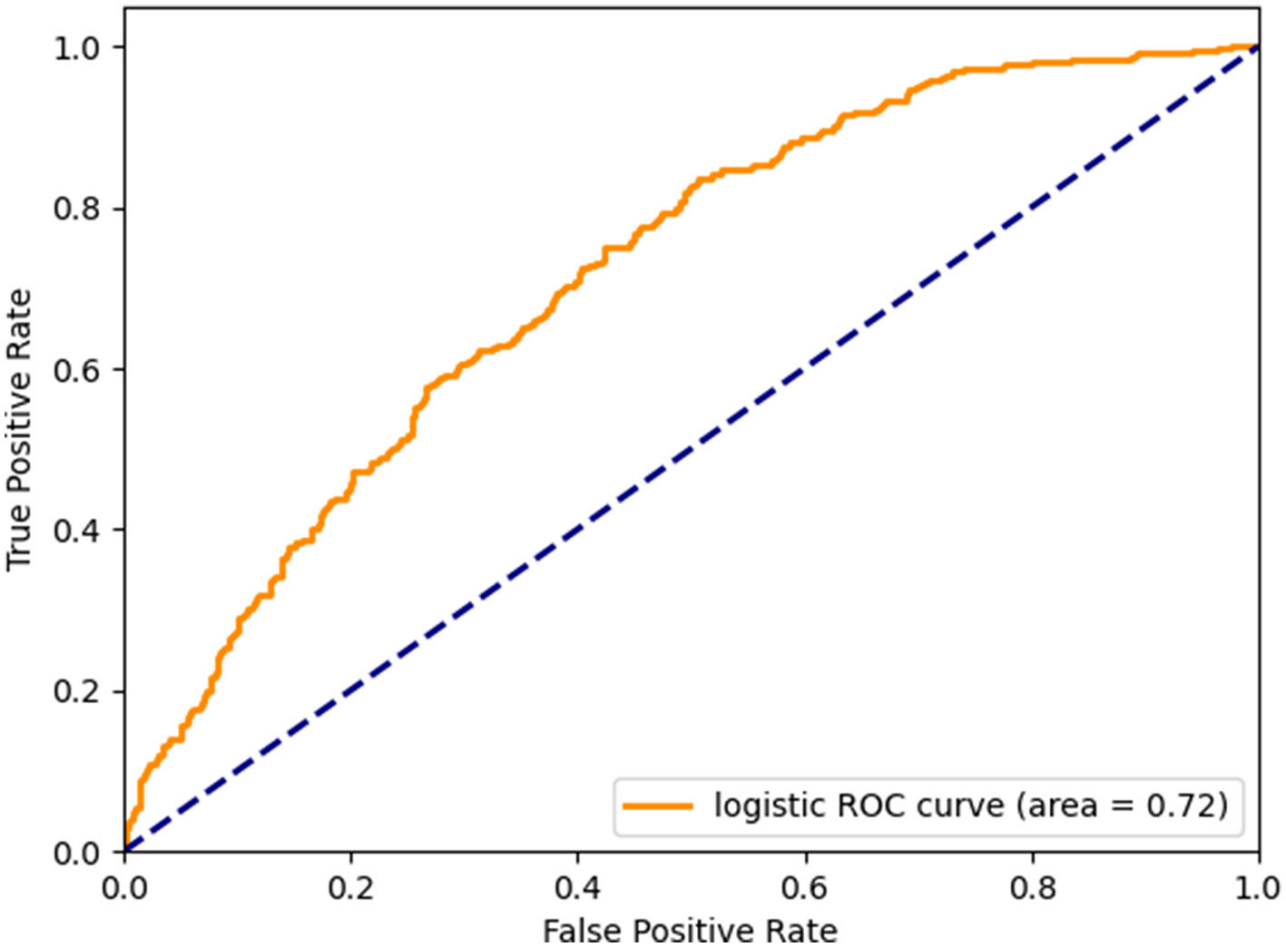
Micro-mean Receiver Operating Characteristic curves plotted according to multivariate logistic regression.

Following the construction of the multivariate logistic regression model, the receiver operating characteristic curve (ROC) was generated for validation (Figure 3). The findings indicated that the area under the curve (AUC) value of the ROC curve for the multivariate logistic regression model was 0.72, indicating the significant clinical relevance of 25-(OH)D in the diagnosis, treatment, and prognosis assessment of cognitive impairment. This information holds importance in informing clinical decision-making and enhancing personalized treatment approaches.

### 3.5 Subgroup and sensitive analysis

The potential for reverse causality poses a significant risk in cross-sectional studies. To mitigate this bias, subgroup analyses and interaction tests were conducted to assess variations in the relationship between 25-(OH)D levels and cognitive frailty across different demographic variables such as sex, age group (65–72 and 73–80 years), education, race, and marital status (27). The findings revealed no significant interactions among the subgroups, except the 65–72 age group where a notable interaction was observed between 25-(OH)D levels and the risk of cognitive frailty (Table 4).

**Table 4:** Subgroup analysis and interaction results.

**Variables**		**Coefficient**	**OR**	***P* interaction**
Education				**0.7**
	Primary education and below		***Reference***	
	Secondary education	0.001	1.0 (0.8, 1.3)	0.99
	Higher education	0.09	1.1 (0.8, 1.4)	0.5
Marital status				**0.1**
	Married		***Reference***	
	Unmarried	0.1	1.1 (0.7, 1.9)	0.6
	Divorced	0.3	1.3 (1.0, 1.8)	0.08
	Widowed	−0.1	0.9 (0.7, 1.1)	0.3
Race				**0.2**
	Asian		***Reference***	
	African-American	0.2	1.3 (0.8, 2.0)	0.3
	The White race	−0.05	1.0 (0.6, 1.4)	0.8
	Latino/Hispanic	0.2	1.3 (0.8, 2.0)	0.4
	Other race	−0.4	0.7 (0.3, 2.0)	0.5
Age				**0.05**
	Age (73–80)		***Reference***	
	Age (65–72)	−0.2	0.8 (0.7, 1.0)	0.05
Gender				**0.8**
	Male		***Reference***	
	Female	−0.03	1.0 (0.8, 1.2)	0.8

This outcome reinforces the applicability of population-based results across various subgroups, underscoring their coherence and dependability. Subsequently, we conducted sensitivity analyses to validate the robustness of the results. By categorizing 25-(OH)D into three groups - deficient, insufficient, and normal - and reevaluating the data, we found results consistent with the analysis in Supplementary Table 2, affirming an inverse relationship between 25-(OH)D levels and cognitive frailty.

### 3.6 Importance of variables

**Figure 4:**
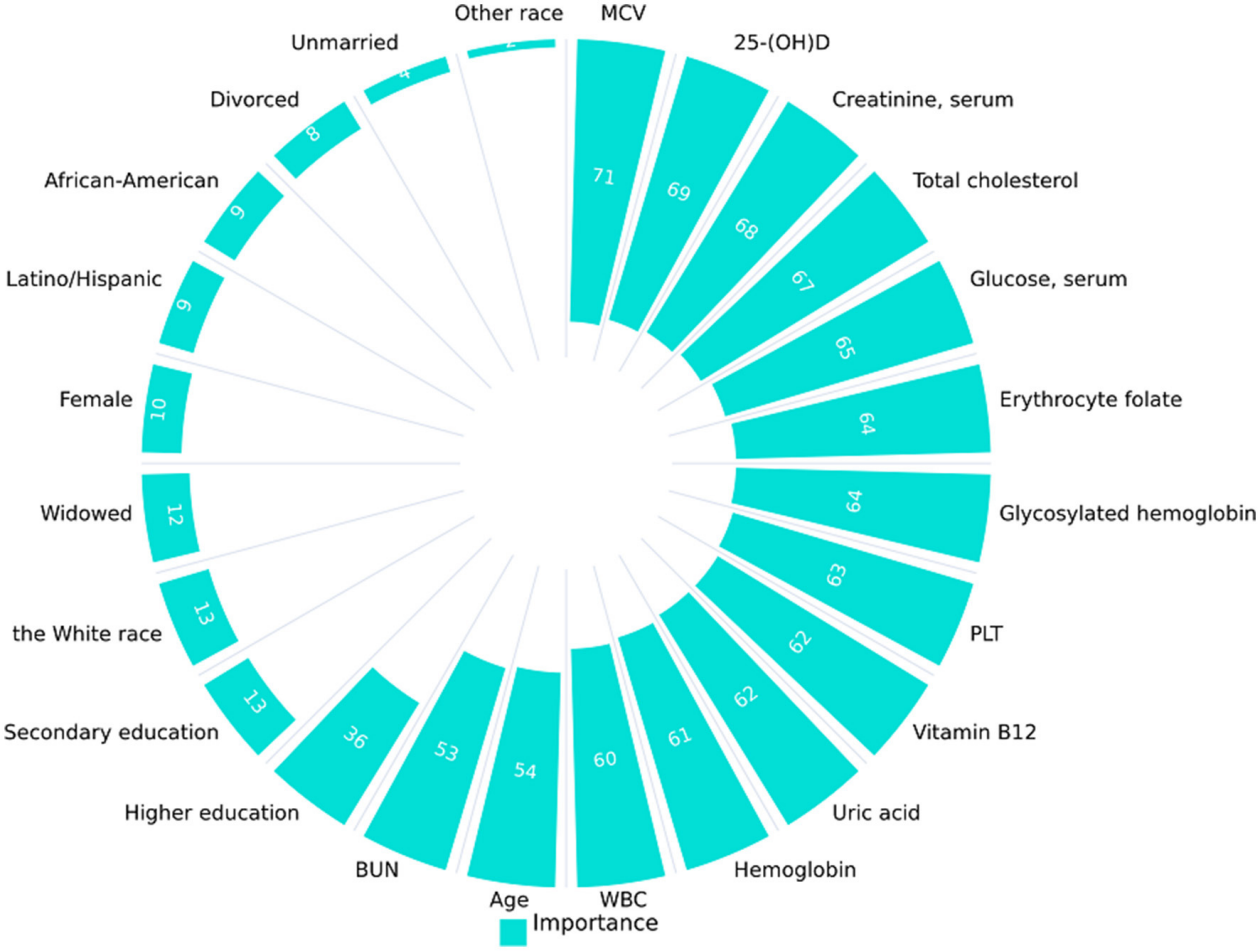
Variable importance ranking.

In the developmental and validation stages of the study, all variables were inputted into a random forest machine learning algorithm utilizing a 10-fold cross-validation methodology to determine relative importance rankings (Figure 4). In this process, we initially load and clean the data. Subsequently, the variables are preprocessed by categorizing them into continuous and categorical types. Following this, the data is partitioned into training and test sets. Feature selection is then conducted using recursive feature elimination and cross-validation (RFECV) within the framework of a logistic regression model. RFECV assesses the importance of each feature and iteratively eliminates the least significant ones. Finally, the selected features and the cross-validation scores from each stage of the recursive feature elimination process are documented, and the identified features are preserved. This methodology enables RFECV to autonomously determine the most significant features, thereby streamlining the model and enhancing its predictive accuracy.

This was done to gain insight into the impact of these variables on cognitive frailty and to support future research and the development or modification of interventions. Furthermore, given that these variables encompass the socio-demographic data of the participants as well as all pertinent laboratory data, the results may be subject to some degree of uncertainty due to the lack of adjustment for potential confounding factors.

## 4 Discussion

The co-occurrence of physical frailty and cognitive impairment, known as cognitive frailty, has been linked to increased susceptibility to negative health consequences such as mortality, disability, hospitalization, and the onset of dementia (28). The etiology of cognitive frailty remains unclear, however, various risk factors such as sociodemographic factors, social status, nutritional status, physical and cognitive activity, and physiological functioning have been identified as strongly correlated with cognitive frailty. These findings have prompted the development of exercise rehabilitation and nutritional therapy as primary therapeutic interventions (4, 28). Prior research has established a strong correlation between 25-(OH)D, vitamin B12, and frailty. Nevertheless, the specific relationship between 25-(OH)D, vitamin B12, and subtypes of frailty, particularly cognitive frailty, remains inadequately understood. This study employs data from the NHANES to investigate the potential protective effects of 25-(OH)D and vitamin B12 in mitigating cognitive frailty among elderly individuals. The findings suggest that 25-(OH)D may play a significant role in protecting older adults against cognitive frailty, whereas vitamin B12 is not.

Several theories may explain our findings. Specifically, 25-(OH)D may enhance cognitive function through mechanisms including neuroprotection, modulation of oxidative stress, regulation of calcium homeostasis, and inhibiting inflammatory processes (29). For instance, in a cross-sectional study involving 2273 older adults, it was observed that individuals with serum 25-(OH)D levels exceeding 20 ng/mL exhibited elevated cognitive scores and a reduced likelihood of cognitive impairment. Conversely, those with serum 25-(OH)D levels equal to or below 20 ng/mL demonstrated a 1.6-fold increased risk of cognitive impairment compared to those with levels exceeding 20 ng/mL (30). Additionally, 25-(OH)D has been shown to modulate cognitive function development through its interaction with the 25-(OH)D receptor (VDR), a nuclear hormone receptor present in the central nervous system and widely distributed among various neurons and glial cells (31). Studies have further demonstrated a notable resemblance in the distribution of VDR between humans and rodents, particularly in regions such as the hippocampus, cerebral cortex, and limbic system, which underscores the significance of 25-(OH)D in the modulation of cognitive functions, including learning and memory (32, 33).

In addition, vitamin B12 (cobalamin), plays an important role in the normal functioning of the brain and nervous system through its association with the cellular metabolism of carbohydrates, proteins, and lipids, and as a cofactor in myelin formation and the normal physiology of the nervous system. Vitamin B12 deficiency has been linked to a range of severe neuropsychiatric symptoms, including depressive symptoms, suicidal behavior, mania, psychosis, and cognitive decline (34, 35). Research has demonstrated a notable correlation between brain size and vitamin B12 levels in individuals aged 61–87 years, with low cobalamin levels increasing the likelihood of cognitive decline, dementia, and Alzheimer's disease, as well as a 5-fold increase in the rate of brain atrophy (36). Additionally, a prospective case-control study found that vitamin B12 supplementation led to significant improvements in frontal lobe function among patients experiencing cognitive decline (37). While previous studies have indicated a potential link between cognitive frailty and vitamin B12 levels, our research indicates that vitamin B12 is not a standalone risk factor for cognitive frailty. This is similar to the findings of a rigorous meta-analysis that investigated the impact of vitamin B12 on cognitive function, depressive symptoms, and fatigue in individuals without advanced neurological conditions. The ongoing debate regarding the association between vitamin B12 and cognitive frailty notwithstanding, the aforementioned results and underlying pathophysiological mechanisms provide a rational explanation and substantiate the assertion that 25-(OH)D exerts a protective influence on the progression of cognitive frailty. These findings hold potential implications for clinical practice aimed at enhancing the outcomes of individuals with cognitive impairment. Furthermore, subgroup and interaction analyses have bolstered the validity and reliability of our findings.

Furthermore, the potential effects of lipids warrant attention. As shown in Figure 4, total cholesterol (TC) exhibited a particularly strong association with frailty among all the variables examined. TC encompasses various components, including high-density lipoprotein-cholesterol, low-density lipoprotein-cholesterol, and free cholesterol, which collectively reflect the body's overall lipoprotein levels. Although TC and LDL-C levels often exhibit a parallel relationship, it is crucial to prioritize LDL-C as the primary metric when assessing frailty, particularly in the context of potential cardiovascular disease implications. Despite data limitations precluding the inclusion of HDL-C and LDL-C in the study, this limitation was partially mitigated by considering hyperlipidemia and cardiovascular disease when evaluating frail conditions. Future prospective studies should account for the potential impact of dynamic changes in LDL-C levels on the degree of frailty. Another noteworthy finding is that, although our study did not identify vitamin B12 as an independent risk factor for cognitive frailty in the elderly population, there is reliable evidence indicating that the cholesterol-vitamin B12 nutritional pattern is associated with mild cognitive impairment (MCI) and exhibits beneficial effects on MCI (38). Despite the underlying pathological mechanisms remaining poorly understood, the conclusion that the cholesterol-vitamin B12 nutritional pattern can influence cognitive impairment is highly informative for our research team in the design of further study.

The above outcomes suggest that regular dietary supplementation with TC and vitamin B12, including the consumption of meat, eggs, and dairy products, appears to be crucial for the elderly population. However, the implementation of this strategy remains contentious. A comprehensive health study conducted in Singapore revealed that higher red meat consumption during midlife is correlated with an elevated risk of cognitive impairment in later years (39). Conversely, substituting red meat with poultry or fresh fish/shellfish was associated with a decreased risk of cognitive decline. A prospective study focusing on the oldest segment of the Chinese population indicated that higher meat consumption was associated with a reduced likelihood of cognitive impairment (40). These inconsistent findings highlight the necessity of considering local conditions when examining the intricate relationship between meat consumption and cognitive function. Future research should be meticulously designed to account for the potential influence of local environmental factors and prevailing dietary patterns.

This study demonstrates certain strengths and innovations in comparison to prior research. Firstly, the study is grounded in a real population study conducted in the United States, encompassing 2,089 older adults aged 65 and above, thereby constituting a cross-sectional study with a substantial sample size. Secondly, the concept of pre-cognitive frailty in the elderly population was introduced to enhance the identification of older adults at risk of developing cognitive frailty. Furthermore, Spearman's rank correlation coefficient was utilized to establish the correlation between variables and cognitive frailty. Subsequently, multivariable logistic regression models combined with RFECV were employed to identify the most significant variables for enhancing model performance. Subgroup analyses and interaction tests were then conducted to validate the findings and broaden their generalizability.

## 5 Limitations

While our study yielded promising and dependable findings, it is important to acknowledge several limitations. The inherent design of cross-sectional studies presents challenges in establishing causality. To enhance the robustness of our conclusions, future research should consider incorporating prospective cohort studies. Furthermore, future studies should explore the longitudinal correlation between 25-(OH)D levels and individual frailty status to support the advancement of personalized intervention strategies. Furthermore, this study exclusively utilized population-based survey data from the United States, thereby prompting inquiries into the applicability of our results to other nations and geographic areas. Additionally, it is pertinent to acknowledge that elderly individuals frequently experience chronic illnesses such as chronic liver disease and chronic kidney disease. While our investigation encompassed hypertension, diabetes, stroke, and heart disease in the evaluation of frailty, certain disease biomarkers were not taken into account and incorporated. Moreover, diet, particularly the consumption of animal-derived foods, exerts a substantial influence on vitamin levels. Future research should meticulously examine the potential impacts of various dietary patterns, with special attention to those characterized by a high prevalence of vegetarian and meat-based foods, on vitamin status.

## 6 Conclusion

In summary, the findings of this cross-sectional study utilizing data from the NHANES database indicate a significant inverse relationship between 25-(OH)D levels and cognitive frailty. Specifically, a one-unit increase in 25-(OH)D levels was associated with a 12% decrease in the risk of cognitive frailty. Acknowledging the existence of this adverse correlation holds significant practical implications for the prevention of cognitive frailty. Monitoring 25-(OH)D levels and administering appropriate 25-(OH)D supplementation to patients experiencing cognitive frailty also bears important clinical implications.

## Data Availability

Publicly available datasets were analyzed in this study. This data can be found here: the two cycles (2011–2012, 2013–2014) from National Health and Nutrition Examination Survey (NHANES) (https://wwwn.cdc.gov/nchs/nhanes/index.htm).
