# Supplementary material for: Cognitive frailty in relation to vitamin B12 and 25-hydroxyvitamin D in an elderly population: a cross-sectional study from NHANES
Source: Front Nutr. 2024 Aug 27;11:1430722. doi: 10.3389/fnut.2024.1430722 (PMC11383760; doi:10.3389/fnut.2024.1430722)
Supplement: Supplementary file 1 [file Table_1.DOCX]

| **41-item Frailty Scale** | |
| --- | --- |
| Difficulty bathing or standing for long periods | No difficulty = 0;  Difficulty but still able to complete = 0.25  Difficulty and needing help = 0.75  Can not do it at all = 1 |
| Difficulty getting dressed | No difficulty = 0  Difficulty but still able to complete = 0.25  Difficulty and needing help = 0.75  Can not do it at all = 1 |
| Difficulty getting up from a chair | No difficulty = 0  Difficulty but still able to complete = 0.25  Difficulty and needing help = 0.75  Can not do it at all = 1; |
| Difficulty walking around the room or the same floor | No difficulty = 0  Difficulty but still able to complete = 0.25  Difficulty and needing help = 0.75  Can not do it at all = 1 |
| Difficulty eating | No difficulty = 0  Difficulty but still able to complete = 0.25  Difficulty and needing help = 0.75  Can not do it at all = 1 |
| Difficulty bending, kneeling, or squatting | No difficulty = 0  Difficulty but still able to complete = 0.25  Difficulty and needing help = 0.75  Can not do it at all = 1 |
| Difficulty getting in or out of the bed | No difficulty = 0  Difficulty but still able to complete = 0.25  Difficulty and needing help = 0.75  Can not do it at all = 1 |
| Difficulty going up stairs or visiting neighbors | No difficulty = 0  Difficulty but still able to complete = 0.25  Difficulty and needing help = 0.75  Can not do it at all = 1 |
| Difficulty lifting or carrying | No difficulty = 0  Difficulty but still able to complete = 0.25  Difficulty and needing help = 0.75  Can not do it at all = 1 |
| Difficulty shopping | No difficulty = 0  Difficulty but still able to complete = 0.25  Difficulty and needing help = 0.75  Can not do it at all = 1 |
| Difficulty preparing meals | No difficulty = 0  Difficulty but still able to complete = 0.25  Difficulty and needing help = 0.75  Can not do it at all = 1 |
| Difficulty grasping small objects | No difficulty = 0  Difficulty but still able to complete = 0.25  Difficulty and needing help = 0.75  Can not do it at all = 1 |
| Difficulty with housework | No difficulty = 0  Difficulty but still able to complete = 0.25  Difficulty and needing help = 0.75  Can not do it at all = 1 |
| Difficulty with money management | No difficulty = 0  Difficulty but still able to complete = 0.25  Difficulty and needing help = 0.75  Can not do it at all = 1 |
| Difficulty walking medium to long distances | No difficulty = 0  Difficulty but still able to complete = 0.25  Difficulty and needing help = 0.75  Can not do it at all = 1 |
| Exercise consistently or not ever advised by a doctor to exercise | Yes = 0  No = 1 |
| Smoke | Yes = 1  No = 0 |
| Self-assessment of health | Excellent/Very good = 0  Good = 0.25  Fair = 0.5  Bad = 0.75  Poor = 1 |
| Use special equipment to live | Yes = 1  No = 0 |
| Everything was an effort | Not at all = 0  Several times = 0.25  Most of time = 0.75  Almost all the time = 1 |
| Feeling down, depressed, or hopeless | Not at all = 0; Several times = 0.25  Most of time = 0.75  Almost all the time = 1 |
| Feeling happy or feeling good about yourself | Not at all = 0  Several times = 0.25  Most of time = 0.75  Almost all the time = 1 |
| Feeling lonely | Not at all = 0  Several times = 0.25  Most of time = 0.75  Almost all the time = 1 |
| Memory-related disorders | Yes = 1  No = 0 |
| Hypertension or taking related medications | Yes = 1  No = 0 |
| Heart diseases or taking related medications | Yes = 1  No = 0 |
| Coronary heart disease or taking related medications | Yes = 1  No = 0 |
| Stroke or taking related medications | Yes = 1  No = 0 |
| Cancer or taking related medications | Yes = 1  No = 0 |
| Diabetes or taking related medications | Yes = 1  No = 0 |
| Arthritis or taking related medications | Yes = 1  No = 0 |
| Chronic lung diseases or taking related medications | Yes = 1  No = 0 |
| Dyslipidemia or taking related medications | Yes = 1  No = 0 |
| Lose all teeth or wear dentures | Yes = 1  No = 0 |
| Oral health self-assessment | Excellent/Very good = 0  Good = 0.25  Fair = 0.5  Bad = 0.75  Poor = 1 |
| Inability to work/Physical disability/Brain damage/Mental retardation | Yes = 1  No = 0 |
| Sleep quality | No insomnia = 0  Insomnia for a few times = 0.25  Can not sleep for most of the time = 0.75  Can not sleep for almost all the time or need medications = 1 |
| MMSE Scale | ≤10 represents 1 point  11-17 represents 0.75 points  18-20 represents 0.5 points  20-24 represents 0.25 points  >24 represents 0 points. |
| PHQ-9 Scale | 0 represents 0 points  1-5 represents 0.25 points  6-10 represents 0.5 points  11-15 represents 0.75 points  ≥16 represents 1 point |
| Body mass index | <18.5 or ≥28.0 = 1  ≥24.0 and <28.0 = 0.5  ≥18.5 and <24.0 = 0 |
| Waist circumference | ≥90 cm for men and ≥85 cm for women represents 1 point |
