# Supplementary material for: Cognitive frailty in relation to vitamin B12 and 25-hydroxyvitamin D in an elderly population: a cross-sectional study from NHANES
Source: Front Nutr. 2024 Aug 27;11:1430722. doi: 10.3389/fnut.2024.1430722 (PMC11383760; doi:10.3389/fnut.2024.1430722)
Supplement: Supplementary file 2 [file Table_2.DOCX]

| **VIF Factor** | **Feature** |
| --- | --- |
| **1.3** | **Age** |
| **1.2** | **Total cholesterol** |
| **1.3** | **WBC** |
| **1.2** | **MCV** |
| **1.3** | **PLT** |
| **2.1** | **BUN** |
| **2.0** | **Glucose, serum** |
| **2.4** | **Creatinine, serum** |
| **2.1** | **Glycosylated hemoglobin** |
| **1.4** | **Uric acid** |
| **14** | **HCT** |
| **1.6** | **Hemoglobin** |
| **1.1** | **Vitamin B12** |
| **1.3** | **Vitamin D** |
| **1.3** | **Erythrocyte folate** |
| **1.8** | **Female** |
| **1.5** | **Secondary education** |
| **1.7** | **Higher education** |
| **1.1** | **Unmarried** |
| **1.2** | **Divorced** |
| **1.3** | **Widowed** |
| **3.1** | **African-American** |
| **3.8** | **the White race** |
| **2.8** | **Latino/Hispanic** |
| **1.2** | **Other race** |
