# Supplementary material for: Cognitive frailty in relation to vitamin B12 and 25-hydroxyvitamin D in an elderly population: a cross-sectional study from NHANES
Source: Front Nutr. 2024 Aug 27;11:1430722. doi: 10.3389/fnut.2024.1430722 (PMC11383760; doi:10.3389/fnut.2024.1430722)
Supplement: Supplementary file 3 [file Table_3.DOCX]

| ***P*-value** | **Variables** |
| --- | --- |
| **0.01** | **Erythrocyte folate** |
| **＜0.001** | **Age** |
| **0.006** | **Total cholesterol** |
| **0.03** | **WBC** |
| **0.3** | **MCV** |
| **0.95** | **PLT** |
| **0.5** | **Glucose, serum** |
| **0.05** | **Creatinine, serum** |
| **＜0.001** | **Glycosylated hemoglobin** |
| **＜0.001** | **Hemoglobin** |
| **0.03** | **Vitamin D (categorized)** |
| **0.8** | **Gender** |
